# Ultrasound Measurement of Skeletal Muscle Contractile Parameters Using Flexible and Wearable Single-Element Ultrasonic Sensor

**DOI:** 10.3390/s20133616

**Published:** 2020-06-27

**Authors:** Ibrahim AlMohimeed, Yuu Ono

**Affiliations:** 1 Department of Systems and Computer Engineering, Carleton University, Ottawa, ON K1S 5B6, Canada; i.almohimeed@mu.edu.sa; 2 Department of Medical Equipment Technology, Majmaah University, Majmaah 11952, Saudi Arabia

**Keywords:** wearable and flexible ultrasonic sensor, single-element ultrasonic sensor, ultrasonic transducer, piezoelectric PVDF film, skeletal muscle monitoring, electrical muscle stimulation, tetanic contraction, fusion index, muscle contractile parameters

## Abstract

Skeletal muscle is considered as a near-constant volume system, and the contractions of the muscle are related to the changes in tissue thickness. Assessment of the skeletal muscle contractile parameters such as maximum contraction thickness (Th), contraction time (Tc), contraction velocity (Vc), sustain time (Ts), and half-relaxation (Tr) provides valuable information for various medical applications. This paper presents a single-element wearable ultrasonic sensor (WUS) and a method to measure the skeletal muscle contractile parameters in A-mode ultrasonic data acquisition. The developed WUS was made of double-layer polyvinylidene fluoride (PVDF) piezoelectric polymer films with a simple and low-cost fabrication process. A flexible, lightweight, thin, and small size WUS would provide a secure attachment to the skin surface without affecting the muscle contraction dynamics of interest. The developed WUS was employed to monitor the contractions of gastrocnemius (GC) muscle of a human subject. The GC muscle contractions were evoked by the electrical muscle stimulation (EMS) at varying EMS frequencies from 2 Hz up to 30 Hz. The tissue thickness changes due to the muscle contractions were measured by utilizing a time-of-flight method in the ultrasonic through-transmission mode. The developed WUS demonstrated the capability to monitor the tissue thickness changes during the unfused and fused tetanic contractions. The tetanic progression level was quantitatively assessed using the parameter of the fusion index (FI) obtained. In addition, the contractile parameters (Th, Tc, Vc, Ts, and Tr) were successfully extracted from the measured tissue thickness changes. In addition, the unfused and fused tetanus frequencies were estimated from the obtained FI-EMS frequency curve. The WUS and ultrasonic method proposed in this study could be a valuable tool for inexpensive, non-invasive, and continuous monitoring of the skeletal muscle contractile properties.

## 1. Introduction

The analysis of the skeletal muscle mechanical characteristics is of great interest for a wide range of medical applications. The measurement of changes in the muscle physical parameters during contractions provides valuable information. For instance, the assessment of muscle helps in investigating the muscle fatigue [1,2], diagnosing the neuromuscular diseases [3,4], evaluating the progression of treatment and rehabilitation [5,6,7], evaluating the efficiency and effectiveness of physical training [6], estimating the injury risks [8,9], and controlling the prosthetic devices [10,11,12,13]. The skeletal muscle is considered as a near-constant volume system; therefore, the changes in muscle length due to contractions are paralleled by the changes in muscle thickness [9,14]. Monitoring of the change in muscle thickness during contractions would be a useful tool to assess the muscle contractile properties [9,15]. Several studies have provided evidence that the relative changes of the muscle contractile parameters were associated with the skeletal muscle atrophy [16,17], the percentage of muscle fiber types [18,19], general and local muscle fatigue [9,20,21,22,23,24,25], and muscle force production [26,27,28]. Various measurement techniques have been explored and employed for the assessment of muscle functions. The common non-invasive methods include surface electromyography (SEMG), mechanomyography (MMG), and ultrasound imaging.

SEMG is a conventional modality to monitor muscle activity and has been extensively used as a gold standard for assessing muscle electrical parameters for decades [29]. The signal of SEMG represents the sum of the electrical activities generated by the muscles located near the biopotential electrodes attached to the skin surface. The increase of contraction force is a result of the increase of the motor unit recruitment and the firing frequency of activated motor units [30]. The quantification of the muscle mechanical parameters from the SEMG signal has some limitation due to the relationship complexity between the biological electrical signal and the mechanical activity of the muscles, especially for a deeper muscle as the SEMG detects the electrical activity of muscles located near the electrodes [29,31,32]. SEMG measures the muscle electrical activity, but might not provide the direct measurement of the muscle mechanical function [33].

MMG is the measurement of the muscle mechanical activity at the skin surface that results from the voluntary or electrically evoked contraction [33,34,35]. There are several types of sensors employed in the MMG measurement technique such as: piezoelectric sensors, microphones, accelerometers, and laser distance sensors [9,35,36,37]. Piezoelectric contact sensors and microphones are mechanically coupled to the skin surface near the muscle of interest, usually using an elastic band or external support to measure skin surface displacement or vibration [38,39,40]. Accelerometers detect the local motion at the skin surface due to the muscle contraction by measuring the acceleration in one or more axes of the motion directions [41,42,43,44]. Laser displacement sensors (LDS) are a non-contact sensor that measures the distance between the laser-beam head and the skin surface above the muscle of interest [36]. LDS allows a high-resolution measurement of the skin surface displacement due to the muscle contraction [45,46,47,48]. In the MMG measurement, the motion artifacts could be resulted from the sensor lateral displacement at the skin surface and/or the bulk motion of the limb/body where the MMG sensors are attached [49,50]. In addition, the investigations of MMG measurement reliability were reported by [42,43,51,52,53,54,55].

As one of the MMG methods, tensiomyography (TMG) was developed to quantify the muscle contractile properties by measuring the muscle radial displacement during the electrically evoked muscle contraction [15]. The TMG performs the examination of the electrically evoked contractions and exclusively through isometric contraction [56]. The TMG method employs a high-precision displacement sensor. The displacement sensor tip is pressed onto the skin surface with a controlled pre-tension while the sensor body is attached to a fixed support [18]. TMG sensor measures the displacements of the skin surface due to the electrically evoked muscle twitch. Thus, the TMG directly measures the skin surface displacement [57]. The displacement–time curve could be further analyzed to extract the contractile parameters such as maximum muscle displacement, contraction time, sustain time, and relaxation time. During the TMG measurement, the subject is required to be still on the examination table to minimize the limb or body movements in order to reduce the motion artifacts in the measurement [58]. The TMG has gained popularity in recent years as a quantitative and consistent tool for the assessment of the muscle contractile properties among the other MMG methods [9,59].

Ultrasound could provide real-time dynamic images of the internal tissue structure, including skeletal muscles, non-invasively with high spatial and temporal resolutions [60]. This allows a measurement of the internal tissue thickness even for deeper muscle [61,62]. Ultrasound imaging has been widely used in the assessment of skeletal muscle function and tracking the muscle thickness changes during static and dynamic contractions [61,63,64,65,66,67,68,69,70,71,72,73,74,75,76,77]. These studies were based on B-mode ultrasound images captured by a clinical ultrasound imaging system with an ultrasound imaging probe composed of multiple-element piezoelectric ultrasonic transducer (UT). A bulky size of the ultrasonic probe may cause undesired motion over the skin surface during the muscle contraction, especially in that involving limb movement [65,69,72,78]. Such an undesired probe motion could cause an inaccurate estimation of the underlying tissue thickness. A special zig or holder was often used to attach the ultrasonic probe steadily on the desired location to monitor the muscle of interest. However, the pressure exerted on the skin surface by the ultrasonic probe weight and/or the attachment method would restrict the underlying tissue’s natural motion and muscle contraction [79].

Instead of B-mode ultrasound imaging, A-mode ultrasound measurement using a single-element UT made of piezoceramic was proposed as a cost-effective and wearable alternative for the ultrasonic measurement of the muscle thickness changes [80]. A-mode measurement is one-dimensional along the tissue depth and capable of measuring the tissue thickness with a single-element UT [81,82]. Thus, the A-mode single-element UT can be made into a smaller size, which could make the UT attachment more accessible and stable at the desired location on the skin surface. The work of Guo et al. [80,83] might be one of the earliest studies using a single-element UT for tracking the thickness changes during the skeletal muscle contraction. They demonstrated the tracking of the extensor carpi radialis muscle that controls the movement at the wrist. The ultrasonic measurement in their result showed a higher accuracy of tracking the wrist extension in comparison to the SEMG measurement. The study by Sun et al. [84] showed that the single-element UT, that was developed by Hettiarachchi et al. [85], could effectively assess muscle fatigue from the measured muscle thickness changes. Yang et al. [75,86] presented the finger motion prediction from A-mode ultrasound signals using single-element UTs. These studies demonstrated the feasibility of using the A-mode UTs for tracking the muscle motion and thickness changes during contraction. The single-element UTs employed in the above-mentioned studies [75,80,84,86,87] were made of piezoelectric ceramic which is a rigid and inflexible material. The piezoelectric ceramics such as lead zirconate titanate (PZT) are commonly used as a UT material due to their superior electromechanical performance. Therefore, the issue of inconsistent placement of the single-element UTs to the skin may still persist. Guo et al. [80,83] indicated in their work that the ultrasonic tracking errors increased when the dynamic movement of the wrist joint extension increased from 20 to 50 cycle/min. In addition, Shahshahani et al. [87] noted that the placement of a single-element UT was challenging to track the diaphragm wall motion for the respiratory activity monitoring. The single-element UT could be placed firmly on the skin surface using a body-worn band. However, this approach might introduce inevitable compression to the local muscles and may affect their contraction/relaxation behavior.

Comparing with the rigid UTs, a flexible, thin, and lightweight UT may have an advantage for UT attachment to the skin surface since it could be conformably attached to the curved skin surface. The microelectromechanical system (MEMS) technology has recently emerged for fabricating a relatively flexible UT array for medical imaging. Yang et al. [88] and Wang et al. [89] introduced fabricated piezoelectric micromachined (PMUT) arrays by mounting diced PZT piezoceramic into patterned cavities on a flexible polyimide film. Similarly, Mastronardi et al. [90] and Sun et al. [91] used aluminum nitride (AlN) as a piezoelectric material instated of PZT, to be embedded onto a flexible polyimide substrate, enabling more flexible and thinner PMUT. Singh et al. [92], Sadeghpour et al. [93], and Hu et al. [94] exploited the island-bridge technique by mounting small pieces of PZT elements into a microfabricated array of silicon islands that were connected with flexible polyamide joints. Cheng et al. [95] and Chong et al. [96] fabricated a flexible UT array based on capacitive micromachined (CMUT) using a concave bottom electrode. In addition, Gerardo et al. [97] described a method to reduce the fabrication cost for CMUT using polymeric materials that have the potential of fabricating a flexible CMUT.

Several studies examined the wearable and flexible UTs for tracking the internal tissue motion on human subjects. Lanata et al. [98] presented the monitoring of the heart wall motion using a wearable UT. The piezoelectric transducer was based on the flexible polyvinylidene fluoride (PVDF) polymer, which was integrated into a flexible belt wrapped around the chest. Their results showed the potential for the continuous monitoring of the cardiopulmonary activity. Wang et al. [99] demonstrated the monitoring of blood pressure by measuring the arterial wall motion using a stretchable and thin UT [94]. Their stretchable UT offers the capability of measuring human tissue up to a depth of 4 cm, and they demonstrated the estimation of the blood pressure at the carotid, brachial, radial, and pedal arteries using the ultrasonic wall-tracking technique.

The aim of this study is to investigate the feasibility of the developed single-element wearable ultrasonic sensor (WUS) for assessing the contractile parameters of electrically evoked skeletal muscle in A-mode ultrasound measurement. The WUS was made of polymer piezoelectric PVDF film due to its flexibility and low-cost fabrication process for the WUS. The low-cost and simple fabrication process allows the WUS to be disposable, which could be beneficial for medical applications. Monitoring of the electrically evoked skeletal muscle contractions, tetanic contraction progression, and the extraction of the muscle contractile parameters were demonstrated using the developed WUS.

## 2. Methodology

We have been developing a flexible and wearable ultrasonic sensor made of the PVDF polymer piezoelectric film [100,101]. The PVDF piezoelectric film offers mechanical flexibility, thinness, broadband response, and the close matching acoustic impedance to the biological soft tissue, which are suitable features for an ultrasonic sensor to perform continuous monitoring of the tissue motion. However, the PVDF has inherent disadvantages of the relatively weak piezoelectric effect, low dielectric constant, high dielectric loss, and low electromechanical coupling coefficient, compared to piezoelectric ceramics, which causes a poor signal-to-noise ratio (SNR) of the received ultrasonic signals reflected from deeper tissue boundaries or propagated in thicker tissue. One approach to improve the ultrasonic pulse strength of the PVDF WUS is to employ the multiple-layer PVDF films design [101,102,103]. Hence, for the experiments conducted in this study, we have designed and constructed the WUSs using double-layer PVDF films as described in Section 2.1.

In our previous works, the developed WUS measurement capability was successfully demonstrated in monitoring the tissue thickness changes up to the depth of 34 mm due to the muscle contraction in the ultrasonic pulse-echo (PE) mode [101,104,105,106,107,108]. Furthermore, cardiac motion at the tissue depth of 30 mm was successfully monitored by the WUS [101]. In this study, we would like to investigate the feasibility of the ultrasonic method for monitoring a thicker soft tissue (greater than 100 mm) and assess the skeletal muscle contractile parameters using the developed double-layer PVDF WUSs in the ultrasonic through-transmission (TT) mode.

In the PE mode, ultrasound travels twice the distance (round trip) of the measured tissue thickness since the ultrasound is transmitted and received by the same WUS. While the TT mode uses two WUSs, the ultrasound just travels from the transmitter WUS to the receiver WUS making a single trip. Since ultrasound attenuates during the propagation within the tissue due to the absorption, scattering, and/or diffraction, the TT mode has an advantage for the measurement of a thick tissue as it exhibits a superior SNR in comparison with the PE mode. Indeed, we were not able to observe the target ultrasound signals to measure the total thickness of the lower leg in the PE mode under our experimental configuration described in Section 2.2.

The detail of WUS design and construction is given in Section 2.1. The developed WUSs were attached to the lower leg of a human subject to monitor the gastrocnemius (GC) muscle contraction, as explained in Section 2.2. The experiments were conducted with one healthy human subject (male, age 32) as a feasibility study to verify the proof of concept of the proposed WUSs and measurement method of muscle contractile parameters. The GC muscle was evoked by electrical muscle stimulation (EMS), and the tissue thickness changes were continuously measured in order to estimate the muscle contractile parameters. The subject gave the informed consent for inclusion before participating in the study. The study was conducted in accordance with the protocol approved by the Carleton University’s Research Ethics Board-B (protocol # 10496 12-0382).

### 2.1. Wearable Ultrasonic Sensor

**Figure 1:**
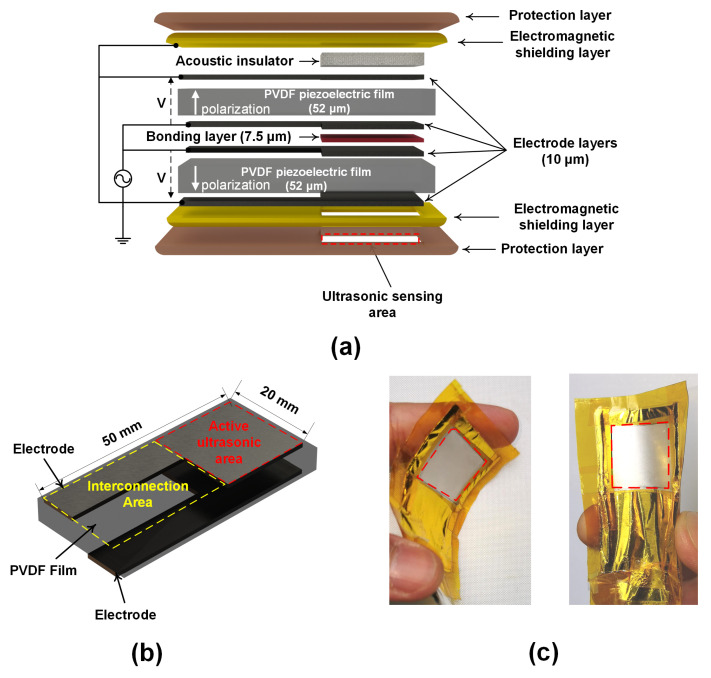
The developed flexible, single-element, wearable ultrasonic sensor (WUS). A schematic design of the WUS (**a**), a schematic of the single polyvinylidene fluoride (PVDF) film with electrodes before the bonding (**b**), and photos of the constructed WUS (**c**).

The WUS was constructed using basic equipment and ordinary hand tools, such as scalpels and soldering device, and did not require advanced technology such as microfabrication processing and equipment used in MEMS-based UT fabrication [88,89,90,92,96]. The piezoelectric PVDF film was chosen to construct the WUS operated in the thickness mode. When an alternating voltage is applied along the thickness direction of the PVDF film, the thickness of the PVDF film increases and decreases periodically according to the frequency of the alternative voltage applied, which will lead to generation of ultrasound waves and vice versa for detection of ultrasound waves [109]. The WUS design consisted of multiple layers, as shown in the schematic of Figure 1a. The WUS had two layers of 52-µm-thick PVDF films as an active piezoelectric element. The PVDF piezoelectric film having silver nanoparticle ink electrodes was obtained from Measurement Specialties (Model: 2-1004346-0, Measurement Specialties Inc., Hampton, VA. USA). The PVDF film was cut into a desired size of the WUS having the active ultrasonic area and the interconnection area, as shown in Figure 1b. The active ultrasonic area is the functional part of the sensor to transmit and receive ultrasound where the electrode layers at the top and bottom surface of the PVDF layer were overlapped. The interconnection area is the part where the lead wires attached to the electrode layers, and there was no overlapping between the electrode layers at the top and bottom surface of the PVDF layer.

The uniformity of the thickness of the silver ink electrode layer was measured 10 ± 1 µm within the active ultrasonic area. The two pieces of the PVDF films were arranged in antiparallel polarization direction and bonded at the inner electrode layers by using a low-viscosity epoxy of 7.5 µm as a bonding layer. Considerations were taken to ensure the uniformity of the epoxy thickness and eliminating the trapped air microbubbles. Both the top and bottom outer electrode layers, next to the acoustic insulator layer and the ultrasonic sensing area, respectively, were connected to the ground electrical terminal. The inner electrode layers, between the two PVDF films, were bonded together and connected to the active voltage source terminal. Thus, the two PVDF films were connected electrically in parallel and acoustically in series with their piezoelectric polarization directions antiparallel. The double-layer PVDF films configuration reduces the input electrical impedance of the sensor, which consequently increases the intensity of the output ultrasonic wave from the available driving voltage source [102]. A flexible brass film for electromagnetic shielding to reduce the environmental noises and a polyimide film for WUS structure protection and electrical insulation were applied to wrap the entire structure of WUS except for the ultrasonic sensing area where the ultrasound is transmitted or received. The electromagnetic shielding and protection layers were removed from the ultrasonic sensing area in order to avoid the ultrasonic attenuation and reflection within these layers. At the backside of the double-layer PVDF films, a piece of paper as an acoustic insulator layer was inserted without bonding between the electromagnetic shielding and the electrode layers. Thus, the WUS was air-backed to eliminate the ultrasonic backward reflection, reduce the sensor thickness, and to improve the electromechanical performance. The developed WUS dimensions were 50 mm × 24 mm with a total thickness of 350 µm. The area of the ultrasonic sensing area was 20 mm × 20 mm. Figure 1c shows photos of the constructed WUS used in this study.

### 2.2. Experimental Configuration

**Figure 2:**
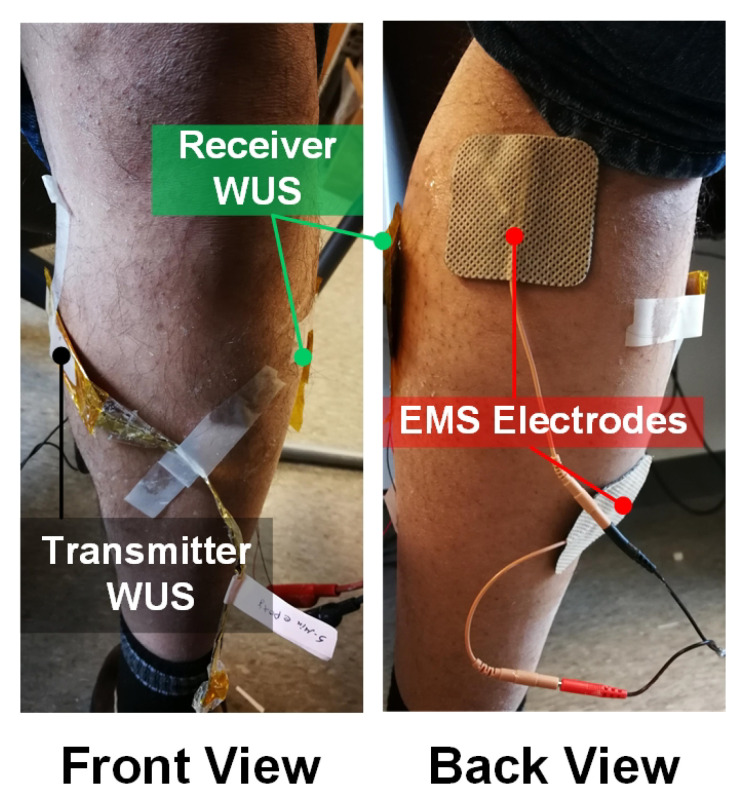
Photos of the experimental setup for monitoring the tetanic contractions of the gastrocnemius (GC) muscle in ultrasonic through-transmission (TT) mode. The two WUSs (transmitter and receiver) and electrical muscle stimulation (EMS) electrodes were attached to the skin surface of a lower leg.

The tissue thickness changes due to GC muscle contraction evoked by the EMS at a lower leg of a healthy male subject were measured using the WUSs in the ultrasonic TT mode. As shown in Figure 2 of the experimental setup, the transmitter WUS was attached to the medial side of the GC muscle at the back of the tibia and fibula bones, whereas the receiver WUS was placed on the opposite side. A medical ultrasonic gel couplant was applied between the skin surfaces and the WUSs. In order to maintain the stability of the WUSs attachment during the measurement, the WUSs and the electric wires were fixed by non-elastic adhesive tapes. Considerations were taken to avoid exerting pressure by the applied adhesive tape so that it would not impede the movements of the underlying tissues including the muscle contraction. The pulsed ultrasound generated by the transmitter WUS propagated through the tissues toward the receiver WUS. The distance, which is the tissue thickness between the transmitter and the receiver WUSs, was estimated by measuring the time-of-flight (TOF) of ultrasound. Average sound speed of 1540 m/s was assumed for soft tissue, including muscles.

**Figure 3:**
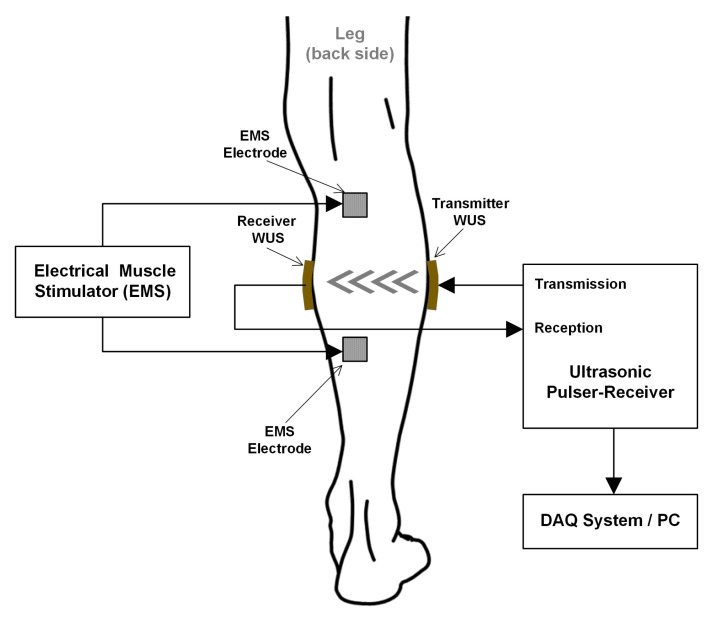
A schematic of the measurement configuration of GC muscle contractions at a lower leg. The muscle contraction was evoked by EMS, and the tissue thickness changes were measured using two developed WUSs in the TT mode.

Figure 3 shows the schematic diagram of the ultrasonic measurement configuration to monitor the electrically evoked GC muscle contraction. The contractions of the GC muscle were evoked by an EMS device (Model: EMS 7500, Compass Health, Middleburg Heights, OH, USA) at EMS frequencies of 2, 6, 8, 10, 12 and 30 Hz with an electric pulse width of 300 µs. The amplitude of the stimulation pulse was adjusted to the comfort level of the human subject depending on the EMS frequency employed.

The WUSs were operated by an ultrasonic pulser/receiver (Model: DPR300, JSR Ultrasonics, Pittsford, NY, USA). The pulse repetition rate (PRR) was 1 kHz, controlled by a function generator. Therefore, one frame of the ultrasonic radio-frequency (RF) signal was acquired every 1 ms. The ultrasonic RF signals received by the receiver WUS were filtered by the pulser/receiver built-in analog band-pass filter of 1–22.5 MHz bandwidth. Then, the received ultrasonic RF signals were digitized at a sampling frequency of 125 MHz and stored by the data acquisition (DAQ) system (Model: ATS 9440, Alazartech, Montreal, QC, Canada) connected to a personal computer (PC). It is noted that the sampling frequency of 125 MHz was the highest of the DAQ system employed, which was much higher than the Nyquist frequency of the received pulsed ultrasound since the real-time measurement was not the focus of this study. In the future study, the lower sampling frequency will be considered and tested for real-time monitoring and signal analysis. The ultrasonic RF signals were acquired for 8 seconds, including a short period without EMS at the beginning of the acquisition at each EMS frequency. Hence, a total of 8000 frames of the RF signals were acquired at each EMS frequency.

**Figure 4:**
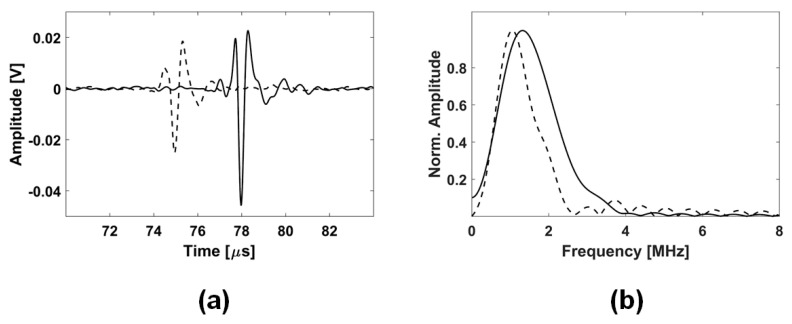
The received ultrasonic radio-frequency (RF) signals using the developed WUSs in the TT mode (**a**) and their frequency spectra (**b**). The dashed line indicates a relaxed state of the GC muscle, where the solid line indicates a contracted state.

For the preprocessing of the acquired ultrasonic RF signals, a moving average of 15 frames was applied to the digitized RF signals to remove the random noise and improve the SNR of the desired signals. The moving averaging of 15 frames at the PRR of 1 kHz was equivalent to the frame rate of 66.6 Hz, which was twice greater than the maximum EMS frequency of 30 Hz in the experiments conducted. Thus, the motion smoothening effect on the measured tissue motion could be negligible. It is noted that no increase of the noise level was observed on the received ultrasonic RF signals after applying the EMS. Figure 4a shows an example of the received ultrasonic RF pulses at relaxed (TOF = 74.95 µs) and contracted (TOF = 77.98 µs) states of the GC muscle indicated by the dashed line and the solid line, respectively. The received ultrasonic pulses were clearly observed in the acquired ultrasound RF signals with high SNR. The TOFs of the ultrasonic pulses were obtained using the peak detection technique of the negative peaks of the received ultrasonic pulses. The tissue thicknesses were calculated as 115.4 and 120.1 mm at the relaxed and contracted states, respectively, using each measured TOF and the assumed ultrasound speed of 1540 m/s. The average center frequency and the bandwidth of the received ultrasonic RF pulses were 1.25 and 1.36 MHz, respectively, as seen in Figure 4b.

## 3. Results and Discussions

### 3.1. Monitoring of Muscle Tetanic Contractions

**Figure 5:**
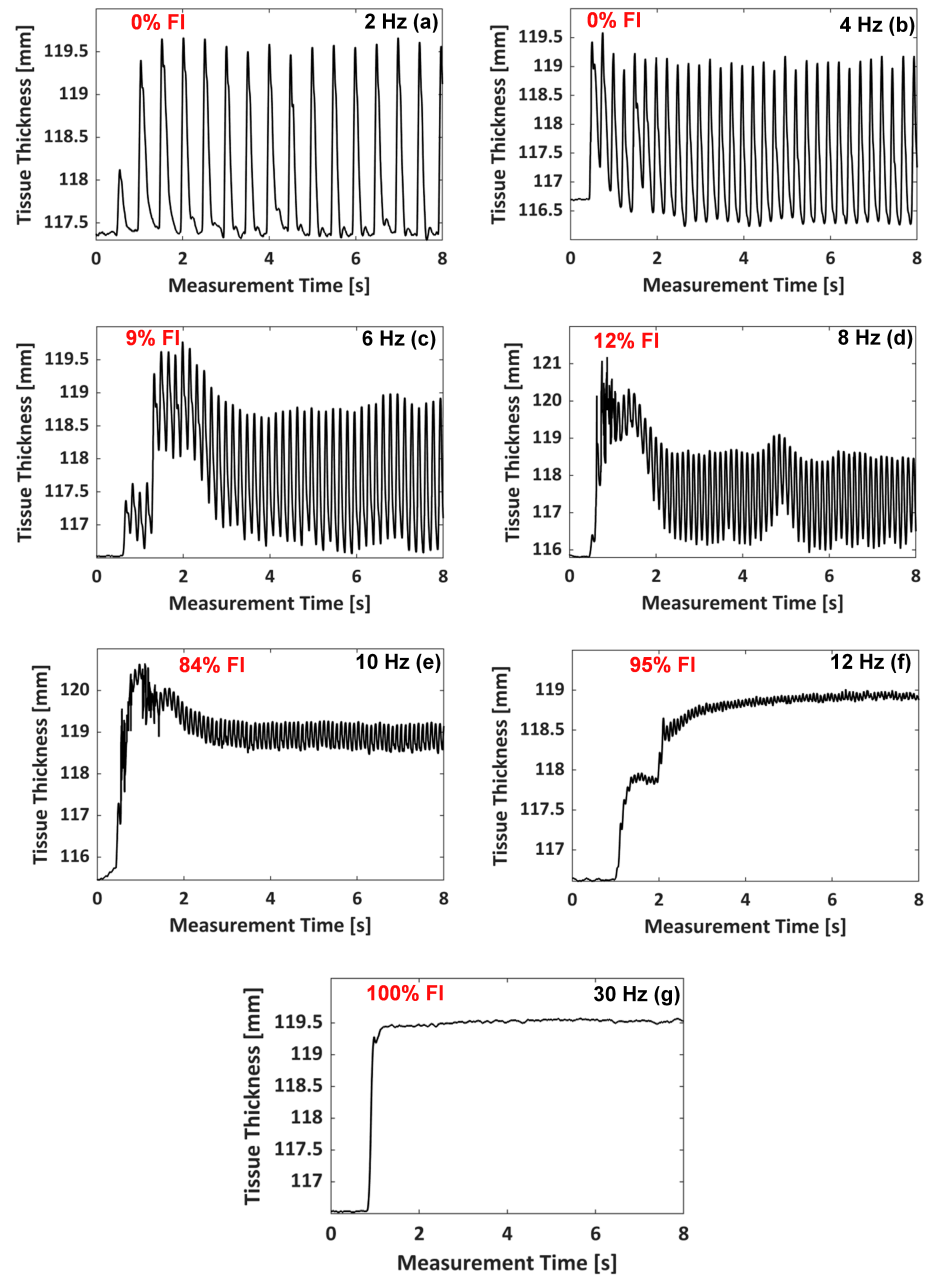
Tissue thickness changes, measured by the WUSs, due to the electrically evoked contraction of GC muscle at EMS frequencies of: 2 Hz (**a**), 4 Hz (**b**), 6 Hz (**c**), 8 Hz (**d**), 10 Hz (**e**), 12 Hz (**f**), and 30 Hz (**g**).

**Figure 6:**
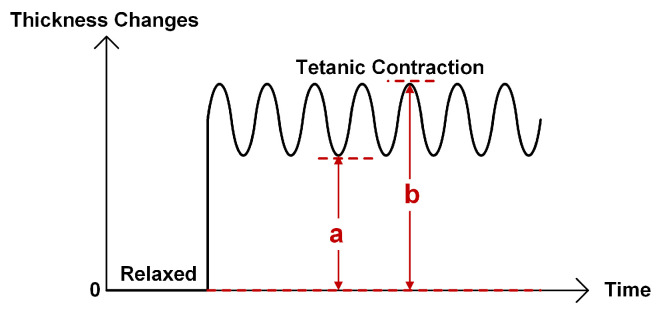
Definition of fusion index (FI) quantifying the tetanic contraction progression level [110,111,112].

Figure 5 shows the changes in tissue thickness obtained during the evoked GC muscle contraction by the EMS at different EMS frequencies. The periodical change of the tissue thickness corresponding to EMS frequency was clearly observed from the GC muscle contraction at the EMS frequency from 2 to 12 Hz. At the EMS frequencies of 2 and 4 Hz, the GC muscle was able to relax completely between the intervals of two consecutive twitches. However, the partial relaxations between the consecutive twitches were seen at the EMS frequencies of 6 to 12 Hz, indicating the progression of tetanic contraction of the GC muscle. No relaxation was observed at the EMS frequency of 30 Hz under our experimental conditions. From the monitored thickness changes at each EMS frequency, the tetanic contraction progression level can be quantified using a fusion index (FI) [110,111,112]. The FI is defined (in %) by:

(1) FI=(a/b)×100

where *a* is the difference between the initial thickness (before the EMS applied) and the minimal thickness during the contraction, whereas *b* is the difference between the initial thickness and the maximal thickness during contraction, as illustrated in Figure 6. Thus, the FI of 0% means the twitch contraction with complete relaxation of the muscle, and that of 100% means the completely fused tetanic contraction.

**Figure 7:**
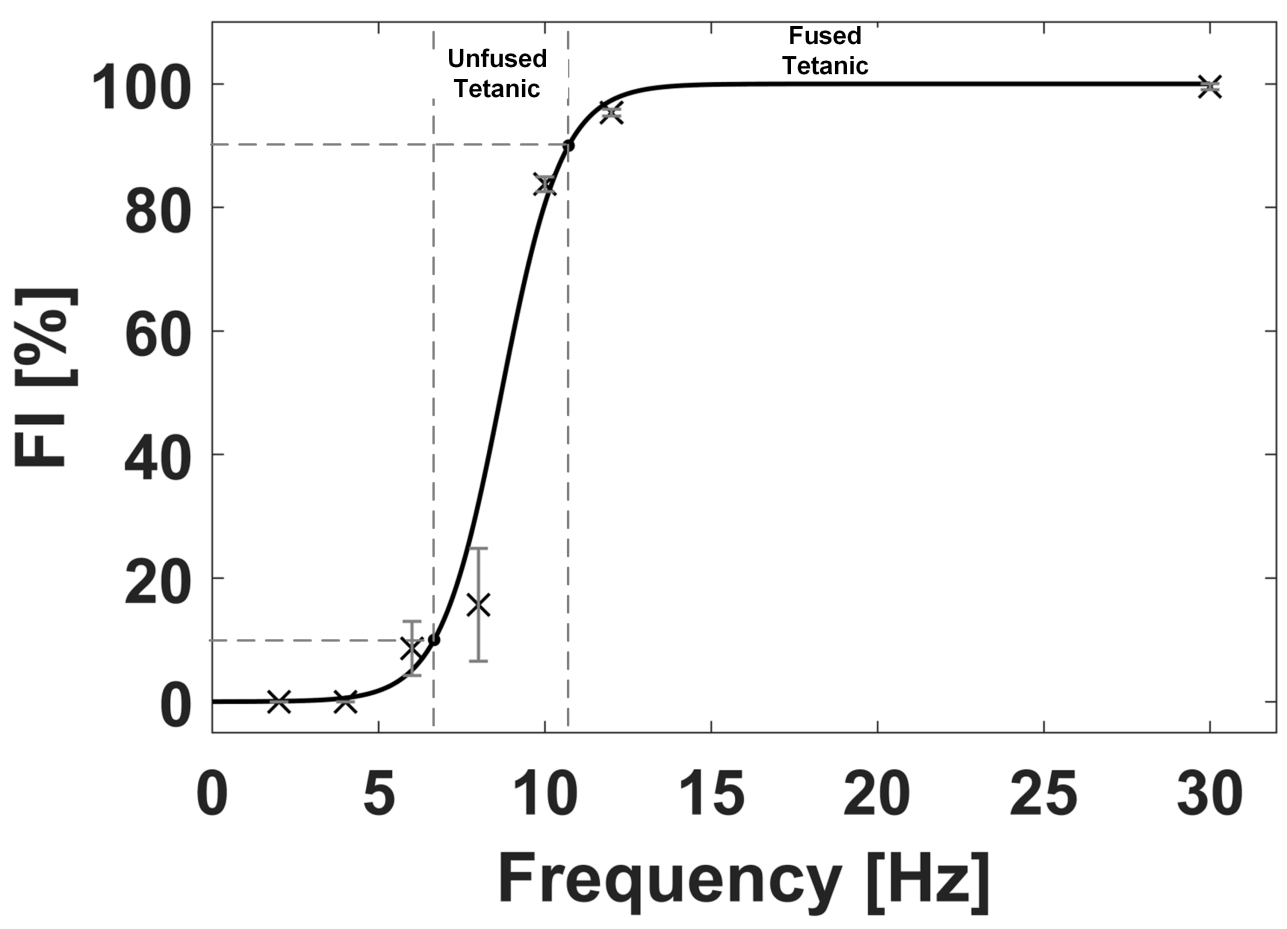
FI value (cross mark) with the standard deviation (error bar) at each EMS frequency, obtained from the results shown in Figure 5. FI-EMS frequency curve denoted by the solid line was obtained by curve fitting of a sigmoid function.

The FI value at each EMS frequency was obtained by taking the average of the calculated FI values between each electrical stimulation interval, after two seconds of initiating the EMS. Figure 7 presents the FI value, denoted by the cross mark, with the standard deviation (SD), denoted by the error bar, obtained at each EMS frequency. As seen in Figure 5a,b at the EMS frequencies of 2 and 4 Hz, respectively, the GC muscle had a complete relaxation between the consecutive stimuli, which indicates the FI of 0%. As the EMS frequency increased to 6 Hz and 8 Hz, the muscle would progress a partial relaxation represented by the FI values of 8.6 ± 4.4% and 15.7 ± 9.1% (mean ± SD), respectively. At 10 and 12 Hz, the tetanic contraction progressed further, and the FI values became 83.8 ± 1.2% and 95.3 ± 0.5%, respectively. The FI value of 100% was assigned at 30 Hz since no muscular relaxation between the consecutive stimuli was observed at 30 Hz, as shown in Figure 5g. In general, the unfused and fused tetanus is considered at FI value greater than 10% and 90%, respectively [113]. Therefore, based on the S-shaped (sigmoid function) curve fitting (solid line) of the obtained FI-EMS frequency relationships shown in Figure 7, the minimum stimulation frequencies necessary to evoke the unfused and fused tetanus were given at FI values of 10% and 90%, respectively, Thus, the GC muscle would be evoked to the unfused and fused tetanic contraction at EMS frequencies of 6.7 and 10.7 Hz, respectively, under our experimental conditions employed.

### 3.2. Muscle Contractile Parameters

**Figure 8:**
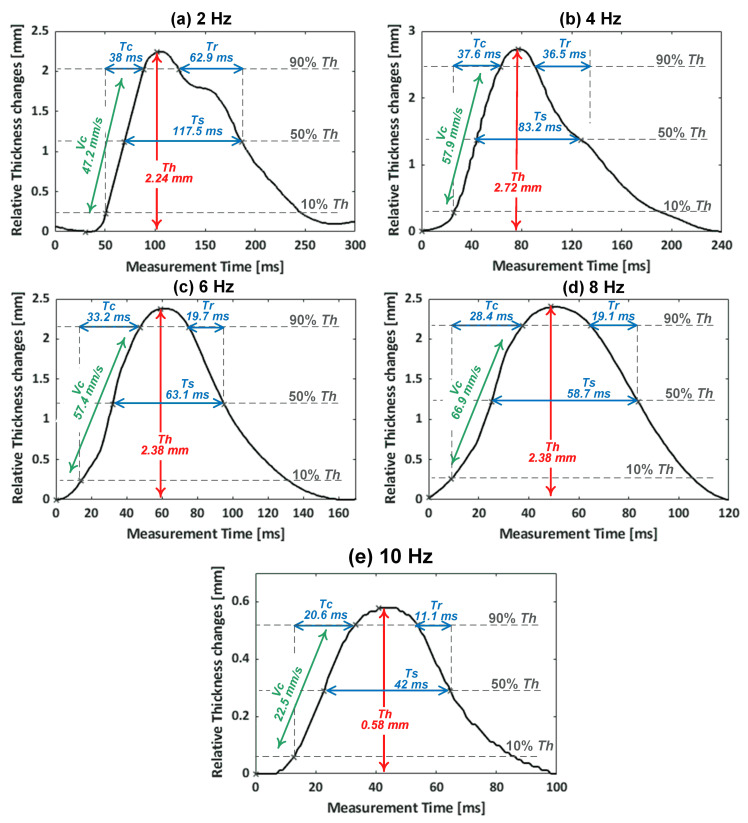
Extracted contractile parameters from a chosen single contraction twitch of the GC muscle evoked at EMS frequencies of: 2 Hz (**a**), 4 Hz (**b**), 6 Hz (**c**), 8 Hz (**d**), and 10 Hz (**e**).

The monitored muscle contractions of the GC muscle were further analyzed by extracting the contractile parameters from a single twitch contraction such as maximum thickness changes, Th, contraction time, Tc, contraction velocity, Vc, sustain time, Ts, and half-relaxation time, Tr [9,15]. Figure 8 shows the extraction of the contractile parameters from a chosen single contraction twitch of the monitored GC muscle at each EMS frequency in the range from 2 to 10 Hz, which were not fused tetanus state in our experiments. At the EMS frequency of 2 Hz, the Th was 2.24 mm from the relaxed state to the maximum contracted state. The Tc during which the thickness increased from 10% to 90% of the Th was 38 ms. During the Tc, Vc was 47.21 mm/s. The Ts during which the GC muscle sustained the 50% of the Th was 117.5 ms. After reaching the maximum thickness, the thickness began to decrease, which indicates the relaxation period. The Tr during which the thickness decreased from 90% to 50% of the Th was 62.9 ms. Table 1 lists the average contractile parameters obtained from the multiple twitches of the electrically evoked GC muscle contractions at each applied EMS frequency, shown in Figure 5. Averaging was taken from the twitches observed after the first two seconds of initiating the EMS, where the twitches became stables.

In Table 1, a coefficient of variation, CV, in percentage (CV = Mean / SD × 100) is also presented for each contractile parameters. The CV values for Th, Vc, and Tc were 8.1% or less, and those for Ts were 11% or less. The CV values for Tr showed greater values (8.3%–21.5%) comparing with the other parameters. It was reported in the TMG measurements with multiple subjects that the Tr parameter exhibited high variability and was indicated as an unreliable parameter, whereas Th and Tc were more reliable parameters with low variability [59]. Though our CV values were estimated from the multiples twitches with only a single subject, a similar tendency of the measurement variability was shown in our results.

Several studies have presented the measurement of the GC muscle contractile parameters [7,16,114,115,116,117,118]. However, the lack of a standardized measurement protocol led to a variety of measurement conditions that limits the comparison between the muscle contractile parameters, as discussed in [59,119,120,121]. The sensor position, EMS conditions (amplitude, pulse width, and inter-electrode distance), and individual differences (gender, age, body mass, and physical activity level) impose difficulties for the direct comparison between the values of the contractile parameters. Therefore, the comparison of the parameter values obtained in this study with those in other studies would not be applicable due to the different measurement conditions employed and is beyond the scope of this study. In future work, we will examine the reliability and repeatability of the WUS measurement method and compare the results with those published values under the same measurement conditions.

**Figure 9:**
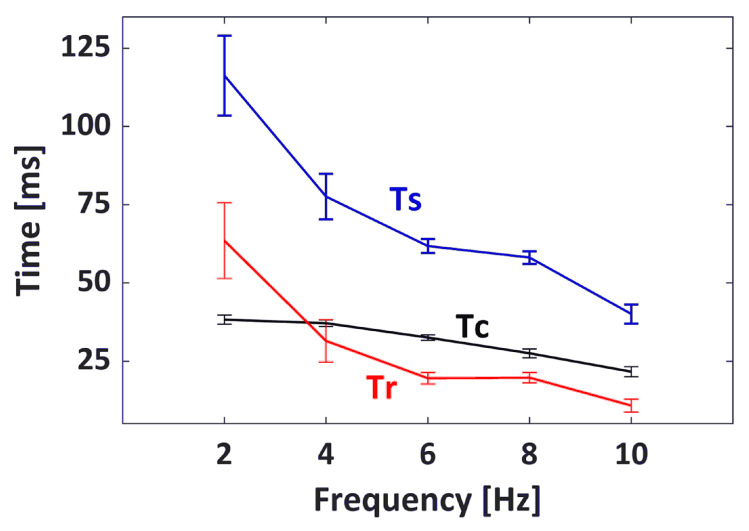
The average contractile parameters of Tc, Ts, and Tr of the monitored muscle contractions with respect to the EMS frequencies. The error bar indicates the standard deviation.

The values of Tc, Ts, and Tr with respect to EMS frequencies from 2 to 10 Hz are presented in Figure 9. It was observed from these extracted contractile parameters that the contraction and relaxation periods of the GC muscle became shorter as the EMS frequency increase. As the EMS frequency increased from 2 to 10 Hz, the average Tc values decreased from 38.25 to 21.62 ms, and the average Tr values decreased from 63.49 to 10.80 ms. In addition, the GC muscle sustained a shorter contraction as the frequency progressed further, which was indicated by the average Ts of 116.28 ms at 2 Hz compared to Ts of 40.07 ms at 10 Hz.

The conducted in vivo monitoring of the tetanic contraction demonstrates the ability of the developed WUS and the ultrasonic method for the continuous and quantitative assessment of the skeletal muscle contractile properties. The novel ultrasonic technique presented in this work allows the measurement of the skeletal muscle contractile parameters beyond the limitations of TMG method, such as fixed posture and/or limited measurement location, which is one of the current popular tools for assessment of the muscle contractile properties [9]. The developed WUS could offer the advantage of the ultrasonic A-mode method by measuring the internal thickness changes of the tissue where the TMG method may not be feasible. The ultrasonic measurement method using the developed WUS could provide a practical alternative with less strict posture conditions, in comparison with the TMG. The proposed WUS and ultrasound method of the measurement of the skeletal muscle contractile parameters would be applied for various medical applications, for instance: monitoring the treatment progression before and after ligament reconstruction surgery [6,7,117], investigation of the fast and slow muscle fibers ratio in muscle [15,16,18], assessment of muscle fatigue [20,122], and physical training effectiveness [9,123]. In addition, the FI-EMS frequency curve would be used to investigate the degree of muscle fatigue [110] and to evaluate the muscle fiber type and fiber composition ratio [111].

In addition, the WUS offers the measurement of both the voluntary and electrically evoked contractions. In previous works, the developed WUS feasibility was demonstrated in monitoring voluntary skeletal muscle contraction [100,101,104,124,125], measurement of arterial diameter [108], and monitoring of mechanical properties of plantar soft tissue [105]. The developed WUS is an inexpensive, flexible, and thin ultrasonic sensor which would provide the ability of the continuous monitoring of the skeletal muscle contractile properties. Additionally, it facilitates the potential of future exploring the skeletal muscle contractile parameters of voluntary contractions during various physical activities.

## 4. Conclusions and Future Perspectives

In this paper, the developed flexible, lightweight, thin, and small WUSs were tested for monitoring the eclectically evoked skeletal muscle contractions of a human subject. The WUSs were designed and constructed using two 52-µm thick PVDF films bonded using a low-viscosity epoxy. The two PVDF films were connected electrically in parallel and acoustically in series with their piezoelectric polarization directions antiparallel for the improvement of the electromechanical performance.

The capability of the developed WUS in measuring the skeletal muscle contractile parameters was demonstrated by the in vivo monitoring of the electrically evoked contractions of the GC muscle at varying EMS frequencies from 2 up to 30 Hz. The tissue thickness changes due to GC muscle contractions were successfully obtained at each EMS frequency by utilizing the ultrasonic TT mode in A-mode data acquisition, where two WUSs employed as transmitter and receiver. The total thickness changes of the tissues between the WUSs due to the muscle contraction were measured using an ultrasound TOF method. The developed WUS was capable of monitoring the progression of the tetanic contraction of the GC muscle at the varying EMS frequencies. The tetanic contraction progression was quantified by the fusion index (FI) estimated from the monitored tissue thickness changes at each EMS frequency. In addition, the fused and unfused tetanus frequencies were estimated from the obtained FI-EMS frequency curve. Furthermore, the muscle contractile parameters such as maximum thickness changes, Th, contraction time, Tc, contraction velocity, Vc, sustain time, Ts, and half-relaxation time, Tr were successfully extracted from the monitored contraction of the GC muscle. As the EMS frequency increased from 2 to 10 Hz, it was observed that the duration of Tc, Ts, and Tr decreased.

This paper demonstrated for the first time, to the best of our knowledge, the monitoring of the tetanic contractions progression of in vivo human skeletal muscle evoked by the EMS using the single-element WUSs in A-mode ultrasonic measurement. It is our expectation that the WUS and ultrasonic method presented in this study would be a valuable tool for inexpensive, non-invasive, and continuous monitoring of the skeletal muscle contractile properties. In addition, it is of our belief that the proposed ultrasound method and the wearability of the developed ultrasonic sensor could have advantages over the conventional methods, such as the reduction of motion artifacts and the reliability during the measurements for both the static and dynamic muscle contractions. Following the encouraging finding presented in this study, the next stage of future work would focus on the reliability and repeatability investigation of the proposed WUS measurement method in comparison with other conventional methods such as TMG and LDS. In addition, more subjects would be recruited in the future evaluation study of the WUS measurement method of the skeletal muscle contractile parameters.

## Figures and Tables

**Table 1:** Average values with the standard deviation (SD) and coefficient of variation (CV) of the contractile parameters from the monitored tetanic contraction of GC muscle at the EMS frequencies.

EMS	Extracted Parameters Mean ± SD (CV)				
	Th (mm)	Vc (mm/s)	Tc (ms)	Ts (ms)	Tr (ms)
2 Hz	2.20 ± 0.08 (3.6%)	46.13 ± 2.76 (6%)	38.25 ± 1.50 (3.9%)	116.28 ± 12.75 (11%)	63.49 ± 12.12 (19.1%)
4 Hz	2.77 ± 0.09 (3.2%)	59.68 ± 2.05 (3.4%)	37.12 ± 1.05 (2.8%)	77.57 ± 7.29 (9.4%)	31.46 ± 6.76 (21.5%)
6 Hz	2.04 ± 0.15 (7.4%)	50.22 ± 3.68 (7.3%)	32.54 ± 0.88 (2.7%)	61.77 ± 2.23 (3.6%)	19.54 ± 1.81 (9.3%)
8 Hz	2.36 ± 0.19 (8.1%)	68.56 ± 5.47 (8.0%)	27.51 ± 1.41 (5.1%)	58.10 ± 2.00 (3.4%)	19.72 ± 1.64 (8.3%)
10 Hz	0.59 ± 0.03 (5.1%)	22.03 ± 1.58 (7.2%)	21.62 ± 1.58 (7.3%)	40.07 ± 3.07 (7.7%)	10.80 ± 2.08 (19.3%)
